# Multifunctional Integrated Transparent Film for Efficient Electromagnetic Protection

**DOI:** 10.1007/s40820-022-00810-y

**Published:** 2022-02-23

**Authors:** Gehuan Wang, Yue Zhao, Feng Yang, Yi Zhang, Ming Zhou, Guangbin Ji

**Affiliations:** College of Materials Science and Technology, Nanjing University of Aeronautics and Astronautics, Nanjing, 210016 People’s Republic of China

**Keywords:** Reduced graphene oxide, Electromagnetic interference shielding, Flexible, Transparent, Silver nanowire

## Abstract

Silver nanowire (Ag NW) has been considered as the promising building block for the fabrication of transparent electromagnetic interference (EMI) shielding films. However, the practical application of Ag NW-based EMI shielding films has been restricted due to the unsatisfactory stability of Ag NW. Herein, we proposed a reduced graphene oxide (rGO) decorated Ag NW film, which realizes a seamless integration of optical transparency, highly efficient EMI shielding, reliable durability and stability. The Ag NW constructs a highly transparent and conductive network, and the rGO provides additional conductive path, showing a superior EMI shielding effectiveness (SE) of 33.62 dB at transmittance of 81.9%. In addition, the top rGO layer enables the hybrid film with reliable durability and chemical stability, which can maintain 96% and 90% EMI SE after 1000 times bending cycles at radius of 2 mm and exposure in air for 80 days. Furthermore, the rGO/Ag NW films also possess fast thermal response and heating stability, making them highly applicable in wearable devices. The synergy of Ag NW and rGO grants the hybrid EMI shielding film multiple desired functions and meanwhile overcomes the shortcomings of Ag NW. This work provides a reference for preparing multifunctional integrated transparent EMI shielding film.

**Supplementary Information:**

The online version contains supplementary material available at 10.1007/s40820-022-00810-y.

## Highlights

- The reduced graphene oxide (rGO) decorated silver nanowire (Ag NW) films were prepared by spray-coating method.
- A highly efficient conductive network was constructed by Ag NW and rGO, achieving superior electromagnetic interference shielding effectiveness of 33.62 dB with a high transmittance of 81.9%.
- The top rGO layer enables the hybrid film with reliable durability, chemical and thermal stabilities.

**Supplementary Information:**

The online version contains supplementary material available at 10.1007/s40820-022-00810-y.

## Graphical abstract

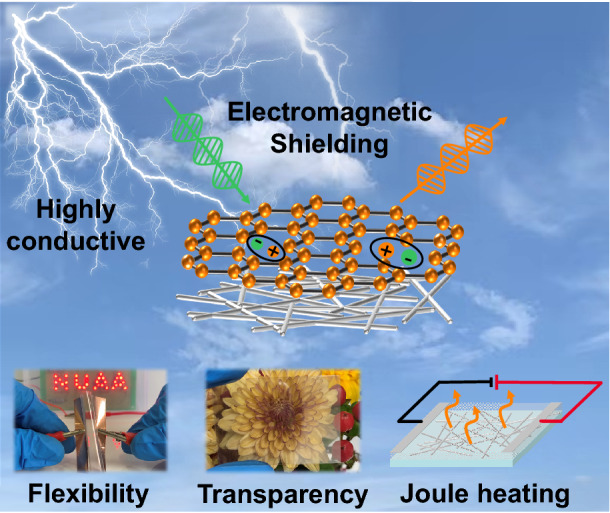

## Introduction

Entering into the age of fifth generation communication, the mushroom development of wireless communication equipment and flexible electronics has accelerated the human society process. However, the accompanying electromagnetic radiation becomes a “health killer” to human [1–4]. Exploring materials with high-efficient and stable electromagnetic wave attenuation capability is a feasible strategy to reduce electromagnetic pollution [5, 6]. Recently, transparent EMI shielding films have sparked huge interest of researchers because these films can meet the requirements of optical observation and electromagnetic protection at the same time [7, 8]. Indium tin oxide (ITO) has been widely used due to its high optical transmittance and good electrical conductivity. However, the inherent brittleness limits its employment in flexible electronics [9, 10]. Ag NW enters the vision of researchers due to excellent electrical conductivity and mechanical flexibility. Moreover, the opening spaces of Ag NW percolation network allow visible light transmission, showing high optical transmittance [11]. For instance, Jung et al*.* proposed a transparent and stretchable Ag NW-based EMI shielding film, which was prepared by deposing Ag NW network on elastic poly(dimethylsiloxane) (PDMS) substrate. The transmittance of the Ag NW/PDMS film reaches 76.8%, and shielding effectiveness (SE) of that is 42.5 dB [12]. Wang et al*.* prepared a transparent Fe_3_O_4_ modified Ag NW EMI shielding film through spin coating method, which exhibits a satisfactory SE of 24.9 dB with high transmittance of 90% [13].

Although high-quality transparent EMI shielding films can be obtained by using Ag NW as building block, there are still some challenges need to be overcome: (i) Ag NW is susceptible to corrosion in air, resulting in EMI shielding performance degradation; (ii) The weak connection between Ag NWs and substrate makes it easy slide and break during bending process; (iii) The insulating capping agent polyvinyl pyrrolidone (PVP) absorbed on the surface of Ag NW and loose contact of adjacent of Ag NWs results in high contact resistance [14, 15]. Introducing a two-dimensional building block as the functional layer is a feasible method to solve these issues mentioned above. For example, Zhang’s group prepared a transition metal carbide/carbonitride (MXene) decorated Ag NW via spray-coating technology. With the help of solvent evaporation induced capillary force, the adjacent Ag NWs can be welded by MXene nanosheets, thus significantly reducing the sheeting resistance (Rs), showing SE about 30 dB with a transmittance of 83%. Besides, the MXene protect layer endows composite film with stable EMI shielding performance after 70 days aging [16]. Liang et al*.* reported a graphene oxide (GO) welded Ag NW transparent conductive film with transmittance of 88% and R_s_ of 14 Ω sq^−1^. After bending 12,000 times with radius of 4 mm, the Rs only increases 2–3% [17]. It was reported that monolayer graphene prepared by chemical vapor deposition (CVD) method exhibits excellent optical transmittance of 97.8%, while the Rs is up to 635 Ω sq^−1^ [18]. The combination of CVD-prepared graphene and Ag NW can realize mutually benefit, showing excellent transmittance of 90% and electrical conductivity of 14 Ω sq^−1^ [19]. Considering the complicated preparation process of MXene or monolayer graphene, and the poor conductivity of GO, rGO is deemed as the ideal material to improve EMI shielding performance and stability of Ag NW-based films. The chemical reduction in GO film usually uses highly poisonous hydrazine or NaBH_4_ [20, 21]. L-ascorbic acid is a nontoxic reducing agent, which reduces GO and does not damage Ag NW network simultaneously.

Herein, we introduce a transparent flexible rGO/Ag NW EMI shielding film, which was fabricated by spray-coating, followed by a mild heating treatment. The rGO nanosheets were uniformly wrapped on the Ag NW network, providing local conductivity for the insulating opening spaces of Ag NW network. Compared with the original Ag NW film, the EMI shielding properties can be enhanced by rGO decoration, whereas the resultant films remain high optical transmittance. Besides, the resultant film can bear 1000 times bending cycles and keep chemical stability for a long time due to rGO protection layer. In addition, the rGO endows the rGO/Ag NW film with exceptional thermal repeatability and stability. These charming features grant the rGO/Ag NW film promising prospects in wearable and optoelectronic application.

## Experimental

### Materials

Ag NW (the diameter and the length are about 30 nm and 20 μm, respectively) suspension in ethanol was purchased from Nanjing XFNANO Co., Ltd. GO aqueous solution was purchased from Institute of Coal Chemistry Chinese Academy of Sciences. L-ascorbic acid was purchased by Sinopharm Chemical Reagent Co., Ltd. All of the materials can be directly used without purification.

### Fabrication of rGO/Ag NW Film

First, the polyethylene terephthalate (PET) substrate was cleaned by absolute alcohol and deionized water for several times and then exposed to UV-ozone atmosphere (SC-UV-I) for 5 min. Subsequently, Ag NW network and GO nanosheets were deposited on the PET substrate in turn by a commercial spray gun (S-130). The pressure and distance were 0.3 MPa and 10 cm, respectively. After drying, the L-ascorbic acid solution was poured into an autoclave, the GO/Ag NW film was immersed in it and heated at 95 °C for 2 h. The mass ratio of GO and L-ascorbic acid was 1:10. Finally, the rGO/Ag NW film was cleaned by deionized water to remove excess L-ascorbic acid, followed by drying. The area density of these building blocks is related to the concentration and volume, which can be expressed by the following equation [22]:

1 $$\sigma_{B} = \frac{{C_{B} \times V_{B} }}{{S_{PET} }}$$

where *σ*_*B*_ represents the area density of building blocks, *C*_*B*_ and *V*_*B*_ are the concentration and volume of building blocks, and the S_PET_ is the area of PET substrate, respectively. The samples were denoted as rGO/Ag NW_x/y_, where x and y represent the area density of rGO and Ag NW.

### Characterization

The micromorphology and elemental composition of the samples were recorded by scanning electron microscope (SEM, Hitachi S4800) and energy-dispersive spectrometer (EDS). The crystal phases of the samples were observed by X-ray diffraction (XRD, Bruker D8 ADVANCE). The binding energy of the samples was recorded by X-ray photoelectron spectrometer (XPS, PHI 5000 VersaProbe). Raman spectra were obtained by Raman spectrometer (Renishaw InVia). The water contact angle was characterized by contact angle meter (SL200B). The transmittance was obtained by UV–visible spectrophotometer (UV-3600). The testing wavelength region ranges from 400 to 800 nm, using neat PET substrate as the baseline. The sheet resistance was recorded by four-point probe measurement (HPS2661). The electrical heating capability of samples was characterized by a DC power supply source (MS-30100) and the infrared (IR) thermal images was recorded by IR camera (FLIR ONE PRO). The electromagnetic parameters, including reflection (S_11_) and transmission (S_12_) parameters, at X band (8.2–12.4 GHz) were recorded by vector network analyzer (Agilent PNA N5244A). The EMI shielding performance can be calculated by the electromagnetic parameters, the equations are as follows [23]:

2 $$R = \left| {S_{11} } \right|^{2}$$

3 $$T = \left| {S_{12} } \right|^{2}$$

4 $$A = 1 - R - T$$

5 $$SE_{T} = SE_{R} + SE_{A}$$

6 $$SE_{R} = - 10log\left( {1 - R} \right)$$

7 $$SE_{A} = - 10\log \left( {\frac{T}{{1 - R}}} \right)$$

where R, A, and T represent reflection, absorption, and transmission coefficients, respectively. SE_T_, SE_R_, and SE_A_ are total, reflection and absorption shielding effectiveness.

## Results and Discussion

### Fabrication and Characterization of rGO/Ag NW Film

**Fig. 1:**
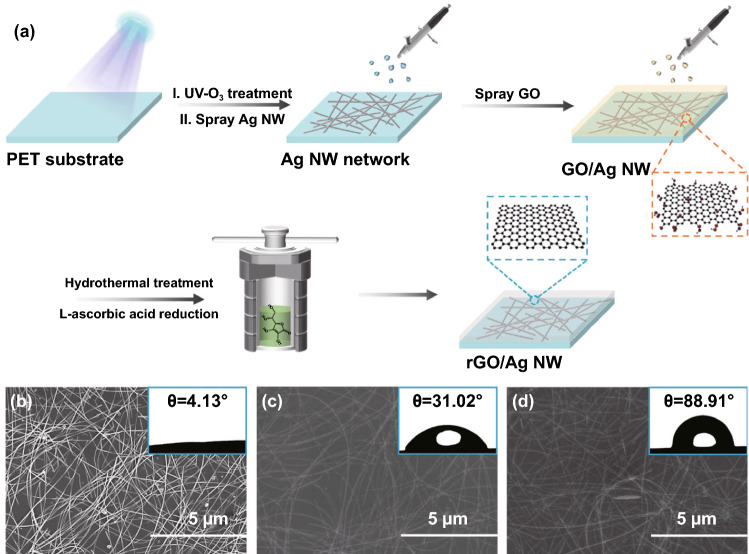
**a** Schematic illustration of the fabrication procedure of rGO/Ag NW film. **b-d** SEM images of the Ag NW, GO/Ag NW, rGO/Ag NW films. The insets are the water contact angle of the corresponding films

The schematic fabrication of rGO/Ag NW film is illustrated in Fig. 1a. First, the UV-O_3_ atmosphere treatment was used to graft hydrophilic groups on PET substrate, which facilitates the uniform deposition of building blocks. Then, the Ag NW network was first deposited on the substrate, and the GO nanosheets were then uniformly covered on the Ag NWs surface, forming a sandwich structure. Finally, the GO nanosheets were reduced by L-ascorbic acid under a mild hydrothermal treatment, forming a highly efficient conductive network. As shown in Fig. 1b, Ag NWs were distributed randomly on the PET substrate. The Ag NW/PET film exhibits hydrophilic character with a water contact angle of 4.13°, which arises from the hydrophilic capping agent PVP absorbed on the surface of Ag NW. Figure 1c displays the Ag NW network wrapped by GO nanosheets, it also shows hydrophilicity (θ = 31.02°) due to abundant functional groups on the surface of GO. After L-ascorbic acid reduction, the Ag NW network maintains integrity. The water contact angle increased significantly (θ = 88.91°), which proved that most functional groups on GO surface have been removed (Fig. 1d). Besides, the EDS spectra proved that GO and rGO are coated on the surface of Ag NW network successfully (Fig. S1).

**Fig. 2:**
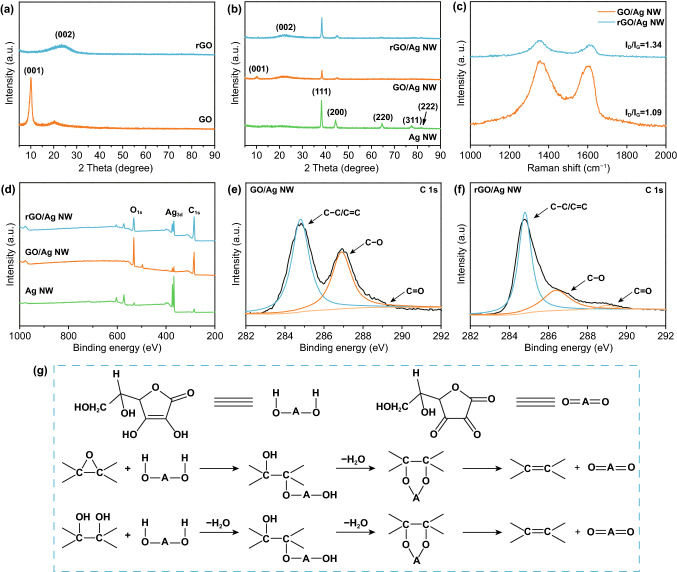
**a** XRD pattern of GO and L-ascorbic acid reduced rGO. **b** XRD pattern of resultant Ag NW, GO/Ag NW and rGO/Ag NW. **c** Raman spectra of the GO/Ag NW and rGO/Ag NW films. **d** XPS spectra of the Ag NW, GO/Ag NW and rGO/Ag NW films. **e**, **f** C 1s spectra of the GO/Ag NW and rGO/Ag NW films. **g** Reaction pathway for the chemical reduction of GO with L-ascorbic acid

For the sake of comparison, we used the same method to prepare rGO nanosheets. As shown in Fig. 2a, the pristine GO exhibits a strong and sharp diffraction peak at 10.2°, corresponding to the (001) plane of GO with interlayer spacing of 8.66 Å. After L-ascorbic acid reduction, the peak at 10.2° disappeared. A broad diffraction peak appears at 23.6°, which is related to the (002) plane of graphite [24, 25]. According to the Bragg's Law, the interlayer spacing of rGO decreases to 3.77 Å. The oxygen-containing groups on the GO nanosheets surface have been removed with the help of L-ascorbic acid, resulting in π-π stacking and graphene aggregation, thus reducing the interlayer spacing of rGO. For the Ag NW network, the XRD pattern shows five typical diffraction peaks centered at 38.1°, 44.3°, 64.5°, 77.4°, and 81.6°, which are indexed to the (111), (200), (220), (311), and (222) crystal plane of face-centered cubic silver, respectively. The XRD pattern of GO/Ag NW shows the typical diffraction peak of GO and Ag NW, proving the successful deposition of GO on the Ag NW network. The chemical reducing agent L-ascorbic acid reduced GO into rGO without damaging the crystal structure of Ag, which reflected on the disappearance of the characteristic peak of GO at 11.1° and the existence of characteristic peaks of Ag NW at 38.1 and 44.3° (Fig. 2b).

As shown in Fig. S2a, both GO and rGO exhibit characteristic peaks of carbon materials, which are located at ca. 1350 cm^−1^ (D band) and ca. 1600 cm^−1^ (G band). The D band is related to defective/disorder carbon, and the G band corresponds to the carbon with sp^2^-hybrid structure [26, 27]. The intensity ratio of D band to G band (*I*_D_/*I*_G_) is used to evaluate the defects degree of carbon materials. The *I*_D_/*I*_G_ value of GO is 1.07, and that of rGO increases to 1.48, which is similar to previous work (*I*_D_/*I*_G_ = 1.51) [28]. The Raman spectra of Ag NW, GO/Ag NW, and rGO/Ag NW are given in Fig. S2b and **2**c. The *I*_D_*/I*_G_ value of the GO/Ag NW is 1.09, and that of the rGO/Ag NW increases to 1.34. The intensity of D band became stronger after reduction, indicating that numerous defects were generated during reduction process, which is beneficial to promote the dissipation of electromagnetic wave [29, 30].

The XPS spectra are shown in Fig. 2d. Both GO/Ag NW and rGO/Ag NW present C 1 s, O 1 s, and Ag 3d peaks. The C 1 s spectra further proved the reduction before and after adding L-ascorbic acid, as given in Fig. 2e–f. The C 1s spectrum of GO can be curve-fit into two main peaks around 284.8 and 286.9 eV, which is related to C–C/C = C and C–O groups [31, 32]. In terms of rGO/Ag NW, the intensity of C–O is dramatically decreased, which conformed that GO has been reduced by L-ascorbic acid.

The chemical reduction mechanism of GO includes two-step S_N_2 nucleophilic and subsequent thermal elimination reactions, as shown in Fig. 2g. The five-membered ring of L-ascorbic acid has a special dienol structure, enabling L-ascorbic acid with reducibility. The hydroxyl groups on the dienol structure are very active, which can dissociate protons and generate ascorbic acid anion (HOAO^−^). During reduction process, HOAO^−^ functions as nucleophilic reagent to attack the epoxy and hydroxyl groups on GO nanosheets. For example, epoxy groups can be opened by HOAO^−^ during a S_N_2 nucleophilic reaction. Then, another HOAO^−^ attacks the hydroxyl groups by a back-side S_N_2 nucleophilic reaction, generating intermediate and H_2_O. Finally, the intermediate can be thermally eliminated, forming reduced graphene oxide. The reduction process of hydroxyl groups is similar to that of epoxy groups [33].

### Optical Transmittance and Electrical Conductivity

**Fig. 3:**
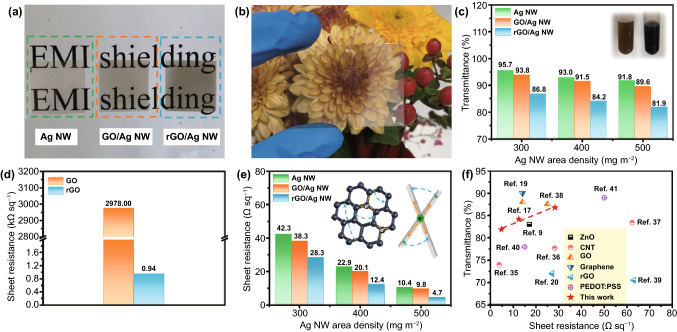
**a** Digital images of the Ag NW, GO/Ag NW, and rGO/Ag NW films. The letters underneath the films are visible. **b** Visual effect of the rGO/Ag NW film. **c** Optical transmittance of prepared Ag NW, GO/Ag NW and rGO/Ag NW films. The insets are the GO (left) and rGO (right) suspension. **d** Sheet resistance of GO before and after reduced by L-ascorbic acid. **e** Sheet resistance of the Ag NW, GO/Ag NW and rGO/Ag NW films. **f** Comparison of sheet resistance-transmittance of the rGO/Ag NW films prepared in this work and other similar transparent EMI shielding films reported previously

For transparent EMI shielding film, optical transmittance and electrical conductivity are critical indicators. As shown in Fig. 3a, single Ag NW network exhibits excellent optical transparency. The GO/Ag NW film shows the typical brownish yellow, which is derived from the color of the GO nanosheets. After hydrothermal treatment, the color of rGO/Ag NW is further deepened but it also has a great visual effect in the range of visible light (Fig. 3b). The optical transmittance of Ag NW, GO/Ag NW, and rGO/Ag NW is given in Figs. 3c and S3. As expected, the transmittance of single Ag NW films decreases with the increase in Ag NW loading density due to the enhancement of photons scattering and reflection [34]. The insets show the color change of GO suspension, and the color of it becomes darker after adding L-ascorbic acid. With the same Ag NW content, the transmittance of the resultant films decreases slightly after covering by GO, and it further decreases after the reduction process. Nevertheless, the optical transmittance remains above 80% at 550 nm.

Figure 3d shows the electrical conductivity of GO before and after reduction. For GO film, the sheet resistance is up to 2918 kΩ sq^−1^, which can be regarded as an insulator, while that of rGO is 0.94 kΩ sq^−1^. The enhancement of conductivity is attributed to the removal of oxygen-containing groups on the GO nanosheets and recovery of graphene π-conjugated structure. Figure 3e compares the sheet resistance of Ag NW, GO/Ag NW, and rGO/Ag NW films. For Ag NW films, the sheet resistance only depends on the area density of Ag NW. The increase in Ag NW content gradually improves the conductive network, providing more paths for electrons transmission, thereby promoting the improvement of conductivity (Fig. S4a–c). The sheet resistance of the GO/Ag NW films is lower than Ag NW films, and it further decreases after reduction treatment. From Fig. S4d–f, it can be seen that the uncovered area reduces with the increase in rGO content. It is sufficient to completely wrap the Ag NW network when the area density of rGO is 50 mg m^−2^. It is obviously that the conductivity of the hybrid films is better than that of the corresponding single Ag NW films. This phenomenon can be explained by the following reasons: (i) The two-dimensional materials have the welding effect on Ag NW junctions due to the capillary force; (ii) The rGO nanosheets fill the uncovered area of Ag NW network, providing local conductivity; (iii) The stacking of Ag NW network and rGO layer can be regarded as two resistors in parallel. The total resistance is lower than Ag NW and rGO, respectively. Sheet resistance versus optical transmittance at 550 nm for rGO/Ag NW films and previous reported Ag NW-based transparent conductive films are plotted in Fig. 3f and Table 1 [9, 17, 19, 20, 35–41]. The rGO/Ag NW films combine the merits of high transmittance and low sheet resistance.

**Table 1:** Sheet resistance and optical transmittance of Ag NW-based transparent conductive films

Materials	T (%)	Rs (Ω sq^−1^)	References
Ag NW/FZO	83	17	[9]
Ag NW/SWCNT	74	3.7	[35]
Ag NW/SWCNT	77.7	28.2	[36]
Ag NW/SWCNT	83.4	62.3	[37]
GO/Ag NW	88	14	[17]
GO/Ag NW	87.6	25	[38]
Ag NW/graphene	90	14	[19]
Ag NW/rGO	72	27	[20]
Ag NW/rGO	70.5	63	[39]
Ag NW/PEDOT:PSS	78	15	[40]
Ag NW/PEDOT:PSS	89	50	[41]
rGO/Ag NW	86.8	28.3	This work
	84.2	12.4	
	81.9	4.7	

FZO: fluorine-doped ZnO; SWCNT: single-walled carbon nanotube; PEDOT: PSS: poly (3, 4-ethylenedioxythiophene): polystyrenesulfonate

### EMI Shielding Performance

**Fig. 4:**
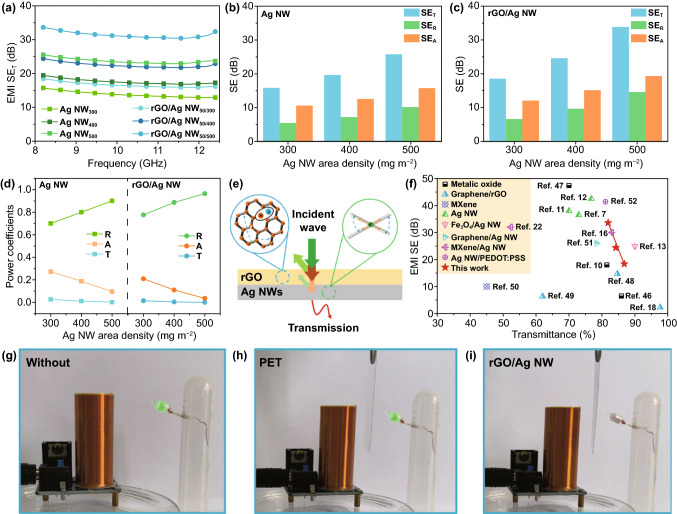
**a** The EMI SE_T_ of the Ag NW and rGO/Ag NW films. **b** SE_T_, SE_R_ and SE_A_ of the Ag NW films. **c** SE_T_, SE_R_ and SE_A_ of the rGO/Ag NW films. **d** Power coefficients of the Ag NW and rGO/Ag NW films. **e** Schematic of the EMI shielding mechanisms of the rGO/Ag NW film. **f** Comparison of optical transmittance-EMI shielding performance of this work with similar work reported recently. **g-i** Digital images of wireless power transfer circuit to demonstrate the EMI shielding application of the rGO/Ag NW film

The rGO/Ag NW film combines high conductivity and excellent optical transmittance, which grants the promising prospects in the field of transparent EMI shielding film. It is well established that the desirable conductivity enables excellent EMI shielding capability [42]. As shown in Fig. 4a, the total EMI shielding efficiency (SE_T_) of the Ag NW films is 15.71, 19.43, and 25.53 dB as the Ag NW area density increases from 300 to 500 mg m^−2^. The welding effect of GO has a small contribution to the improvement of EMI shielding capability, and the resultant GO/Ag NW films show SE_T_ value of 16.75, 20.62, and 27.46 dB (Fig. S5). Compared with the single Ag NW and GO/Ag NW films, the rGO/Ag NW films exhibit strong EMI shielding performance with SE_T_ value of 18.38, 24.38, and 33.62 dB, respectively. These results are consistent with the trend of conductivity.

The SE_T_, SE_A_, and SE_R_ of the Ag NW and rGO/Ag NW films are shown in Fig. 4b–c. The reflection loss is originated from the interaction between carriers and incident EM waves. The higher amount of Ag NWs constructs a perfect conductive network, which provides more transmission path to promote the interaction between carriers and incident EM waves [43]. Similarly, the rGO nanosheets fill the holes of non-connect Ag NWs and provide additional electrical path. Compared with the Ag NW counterparts, both SE_T_ and SE_R_ of the rGO/Ag NW films are higher because of the enhanced conductivity. The Ag NW conductive network is conducive to induced current transmission upon alternating electric field, enabling an electromagnetic-thermal energy conversion [44]. With the increase in Ag NW, the value of SE_A_ shows an upward trend due to the gradually improved conductive network. The introduction of rGO nanosheets constructs a more complete conductive network, which promotes the improvement of SE_A_. In addition, the defects of rGO act as polarization centers, resulting in dipole polarization, thereby enhancing absorption loss [45].

To understand the underlying EMI shielding mechanism, the power coefficients of R, A, and T are plotted in Fig. 4d. With the increase in Ag NW loadings, the value of T changes little, while the value of R shows an obvious upward trend due to the formation of gradually improved conductive network. The R value of rGO/Ag NW is higher than that of the Ag NW, which is related to the enhancement of conductivity. The value of A exhibits a downward trend, and the amount of power shielded by absorption is lower than that of reflection. Generally, the improvement of conductivity also enhances the absorption loss. Here, we introduce the effective absorption coefficient (A_eff_, $A_{eff} = \frac{1 - R - T}{{1 - R}}$) to evaluate the effective contribution of absorption loss, which shown in Fig. S6. As expected, the value of A_eff_ increases with increase in Ag NW content and the introduction of rGO. Overall, the growth of R is greater than that of A. Thus, A shows a downward trend. According to the analysis above, the dominant EMI shielding mechanism of the Ag NW and rGO/Ag NW films is reflection. Based on transmission line theory, the EM wave transfer process is demonstrated as follows (Fig. 4e). First, most incident EM waves were reflected at the shield-air interface because of impedance mismatching. High concentration of carries interacted with EM waves, resulting in strong reflection loss. Second, the electromagnetic energy can be converted into thermal energy via conduction loss and dipole polarization, which contributes to absorption loss. Finally, the residual EM waves continue to transmit through the shield.

To highlight the merits of our work, the comparison of the optical transmittance and EMI shielding performance of this work and similar transparent EMI shielding films are shown in Fig. 4f and Table 2 [7, 10–13, 16, 18, 22, 46–52]. It can be found that the comprehensive performance of rGO/Ag NW films is superior than most of reported literature works. These results indicate that the rGO/Ag NW films are promising candidates for ideal transparent EMI shielding application.

**Table 2:** Comparison of optical transmittance and EMI shielding performance of transparent EMI shielding films

Materials	Substrate	T (%)	SE (dB)	References
ITO	Glass	81.5	~ 18	[10]
Al-ZnO	Glass	85.93	6.5	[46]
Ga-ZnO	Glass	70	47.4	[47]
Monolayer graphene	PET	97.7	2.27	[18]
Multilayer graphene	PET	84.7	14.73	[48]
rGO	PEI	62	6.37	[49]
MXene	Glass	45	10	[50]
PES/Ag NW	PET	70	38	[11]
CA/Ag NW	PU	73	36.5	[7]
Ag NW	PDMS	76.8	42.5	[12]
Fe_3_O_4_/Ag NW	PET	90	24.9	[13]
Graphene/Ag NW	Glass	78.4	26	[51]
Ag NW/MXene	PET	83	~ 30	[16]
Ag NW/MXene/PVA	PC	52.3	32	[22]
Ag NW/PEDOT: PSS	Glass	81.1	41.4	[52]
rGO/Ag NW	PET	86.8	18.38	This work
		84.2	24.38	
		81.9	33.62	

PEI: poly (ethylene imine); PES: poly (ethersulfones); CA: calcium alginate; PU: polyurethane; PVA: polyvinyl alcohol; PC: polycarbonate

A wireless power transmission device was used to demonstrate the EM waves blocking effect of rGO/Ag NW, as shown in Fig. 4g–i and Movie S1. When the power supply is turned on, it can drive the LED bulb to light up whether or not inserting pristine electrical insulating PET substrate. On the contrary, the LED bulb extinguished when inserting the rGO/Ag NW film, suggesting that the wireless transmission has been blocked.

### Mechanical Performance and Chemical Stability

**Fig. 5:**
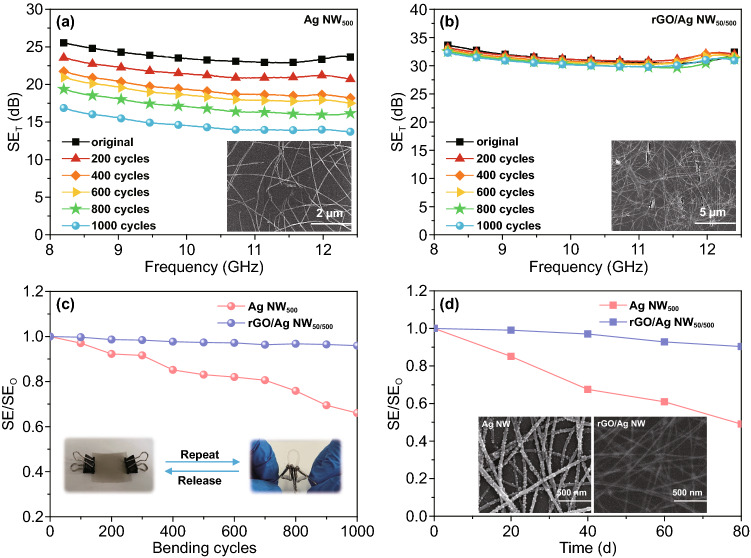
**a**, **b** Change in SE_T_ of the Ag NW and rGO/Ag NW films with various numbers of bending cycles. Insets are SEM images of the Ag NW and rGO/Ag NW films after 1000 times cyclic bending. **c** EMI SE variation in the Ag NW and rGO/Ag NW films during cyclic bending test. The insets are home-made bending test equipment and the bending radius is 2 mm. **d** EMI SE variation in the Ag NW and rGO/Ag NW films during long-time exposure in air at room temperature. Insets are SEM images of the Ag NW and rGO/Ag NW films after long-time oxidation

In addition to high optical transmittance and strong EMI shielding capability, mechanical flexibility and reliable durability are important preconditions for the application in the field of flexible electronics. As shown in Fig. 5a, the EMI SE_T_ value of Ag NW_500_ shows an obvious downward trend during cyclic bending test. Compared with the original, the Ag NW network deforms severely, and most Ag NWs were broken after repeated bending. The conductive network has been destroyed, leading to EMI shielding performance degradation. In contrast, the rGO/Ag NW_50/500_ shows a stable EMI shielding performance under numerous deformations (Fig. 5b). The rGO/Ag NW network presents intact structure, with some cracks on the surface after bending, resulting in EMI shielding performance down slightly. The ratio of SE to original SE (SE/SE_O_) was calculated to evaluate the stability and reliability of repeated straightening-bending cycles. The SE/SE_O_ of the rGO/Ag NW is 0.96, while that of the Ag NW is only 0.66 after bending for 1000 times. (Fig. 5c). Therefore, compared with the Ag NW counterpart, the rGO can bridge the fragmented Ag NW and protect it from slippage, showing a remarkable fatigue resistance upon bending deformation.

The Ag NW is prone to be oxidized if exposed to air. As shown in Fig. 5d inset, numerous oxidized particles appear on the surface of Ag NWs after exposing in air for 80 days. Wrapping by rGO nanosheets, the structure of Ag NW network remains intact for a long time. Changes in EMI SE have been recorded every 20 days to evaluate the chemical stability of Ag NW and rGO/Ag NW film (Fig. 5d). After exposing in air for 80 days, the EMI SE of rGO/Ag NW_50/500_ decreases slightly from 33.62 to 30.37 dB, whereas that of the counterpart declines significantly from 25.53 to 12.51 dB. The rGO coating prevents the direct contact between Ag NW and air, enhancing long-time environment stability.

### Electrical Heating Performance and Thermal Stability

According to our previous work, ice crystals are generated on the surface of PET substrate below 0 °C, leading to unsatisfactory optical transmittance [34]. The electro-thermal ability of transparent EMI shielding films is expected to realize thermal regulation and anti-frozen. In this work, we investigate the surface temperature (T_S_) of the Ag NW and rGO/Ag NW films based on Joule heating effect ($Q = \frac{{U^{2} }}{R}t,$ where Q, U, R, and t represent heat, applied voltage, resistance of EMI shielding film, and time). As shown in Fig. S7a-b, the maximum Ts of Ag NW_300_ and Ag NW_500_ is about 50 °C. It was reported that the melting temperature decreases significantly if the size of Ag reduces to nanoscale. For bulk Ag, the melting temperature is up to 960 °C, while that of the Ag NW is only 200 °C [53, 54]. For Ag NW conductive network, the current is transmitted between nanowires when voltage is applied. Due to the high contact resistance, the generated heat accumulates at the junctions of Ag NW, and the local temperature could reach over 300 °C, resulting in electrical failure [55]. It can be seen that the junctions of Ag NW were fused at high voltages, leading to the fracture of conductive network (Fig. S7c–d).

**Fig. 6:**
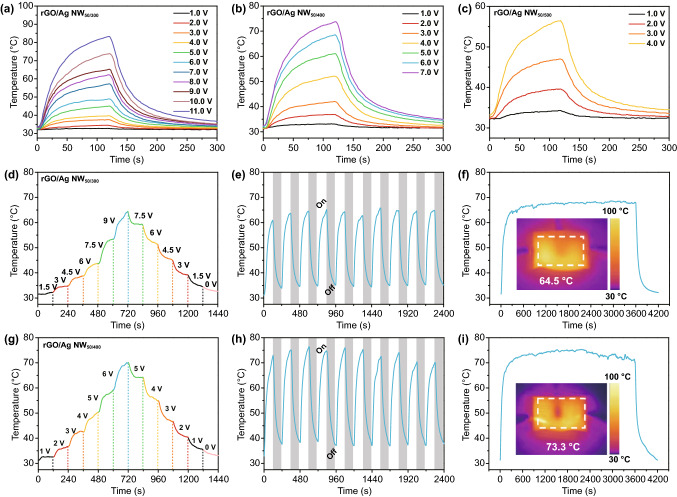
**a–c** Temperature–time profiles of the rGO/Ag NW_50/300_, rGO/Ag NW_50/400_, and rGO/Ag NW_50/500_ at different applied voltages. **d** Tailored surface temperature of the rGO/Ag NW_50/300_ upon stepwise voltages. **e** Heating cycles of the rGO/Ag NW_50/300_ upon repeated applied voltage of 9.0 V. **f** Electrical heating time–temperature curve at a constant voltage of 9.0 V for 1 h. The inset is IR image of rGO/Ag NW_50/300_ at 9.0 V. **g** Tailored surface temperature of the rGO/Ag NW_50/400_ upon stepwise voltages. **h** Heating cycles of the rGO/Ag NW_50/400_ upon repeated applied voltage of 6.0 V. **i** Electrical heating time-temperature curve at a constant voltage of 6.0 V for 1 h. The inset is IR image of rGO/Ag NW_50/400_ at 6.0 V

Figure 6a–c shows temperature–time profiles of rGO/Ag NW films at different applied voltages. The Ts of all specimens increases with the increase in applied voltages. Besides, the films with lower sheet resistance have higher Ts at the same external voltage. For example, at the voltage of 4 V, the Ts of rGO/Ag NW_50/300_ is 39.8 °C, whereas that of rGO/Ag NW_50/500_ is 56.1 °C. In conclusion, an ideal electric heater can achieve high Ts and realize fast temperature response at low driving voltage. Besides, the introduction of rGO reduces the contact resistance and disperses heat into the substrate and air, which is beneficial to improve the thermal stability of rGO/Ag NW. For instance, the highest T_S_ of rGO/Ag NW_50/300_ is up to 83.3 °C, while that of Ag NW_300_ is only 51.6 °C. It should be noted that the highest T_S_ of rGO/Ag NW_50/500_ is similar to the Ag NW_500_, suggesting that a small amount of rGO has little effect on the improvement of thermal stability.

Given the Ts of Ag NW films and rGO/Ag NW_50/500_ can only reach about 50–60 °C, this work focused on the Ts variation in rGO/Ag NW_50/300_ and rGO/Ag NW_50/400_. Figure 6d, g shows the tailored Ts of the rGO/Ag NW_50/300_ and rGO/Ag NW_50/400_ upon stepwise voltages. The real-time temperature ascends or descends with the increase or decrease in operation voltages, indicating thermal regulation capability of rGO/Ag NW films. The repeated heating cycles and long-time heating tests at constant voltage were carried out to estimate the thermal stability and repeatability of rGO/Ag NW films. We chose 9.0 and 6.0 V as the applied voltage of rGO/Ag NW_50/300_ and rGO/Ag NW_50/400_, respectively. Under these voltages, the Ts of rGO/Ag NW films exceeds 60 °C. At the voltage of 9.0 V, the highest temperature of rGO/Ag NW_50/300_ maintains ca. 64.1 °C during repeated on–off cycle heating process (Fig. 6e). Similarly, the highest temperature of rGO/Ag NW_50/400_ maintains ca. 73.8 °C at 6.0 V (Fig. 6h). Figure 6f, i shows the long-time thermal stability of rGO/Ag NW films with Ag NW loading density of 300 and 400 mg m^−2^. The Ts of rGO/Ag NW_50/300_ and rGO/Ag NW_50/400_ fluctuates slightly for heating 1 h, indicating these electrical heaters possess excellent thermal stability. The insets are IR images of rGO/Ag NW_50/300_ at 9.0 V and rGO/Ag NW_50/400_ at 6.0 V, which exhibit uniform temperature distribution.

## Conclusion

In summary, we proposed a facile method, including spray-coating and L-ascorbic acid reduction process, to fabricate flexible transparent rGO/Ag NW films. Compared with the Ag NW films, the introduction of rGO improves the overall conductivity. The resultant rGO/Ag NW film exhibits outstanding EMI SE of 33.62 dB with an optical transmittance of 81.9%. Besides, the EMI shielding performance of rGO/Ag NW has no obvious degradation after 1000 times bending cycles and long-time exposure in air, presenting reliable durability and outstanding stability. Moreover, the transparent EMI shielding films possess the fast thermal response and thermal stability. The integrated multifunction endows the rGO/Ag NW films with huge potential for the application of next-generation flexible and wearable electronics.

## Supplementary Information

Below is the link to the electronic supplementary material.

Supplementary file1 (MP4 2885 kb)

Supplementary file2 (PDF 545 kb)
