# Supplementary material for: Multifunctional Integrated Transparent Film for Efficient Electromagnetic Protection
Source: Nanomicro Lett. 2022 Feb 23;14:65. doi: 10.1007/s40820-022-00810-y (PMC8866598; doi:10.1007/s40820-022-00810-y)
Supplement: Supplementary file 2 — Supplementary file2 (PDF 545 kb) [file 40820_2022_810_MOESM2_ESM.pdf]

Supporting Information for

Multifunctional Integrated Transparent Film for Efficient Electromagnetic Protection

Gehuan Wang[1], Yue Zhao[1], Feng Yang[1], Yi Zhang[1], Ming Zhou[1], Guangbin Ji[1,*]

[1]College of Materials Science and Technology, Nanjing University of Aeronautics and Astronautics, Nanjing 210016, P. R. China

*Corresponding author. E-mail: gbji@nuaa.edu.cn (Guangbin Ji)

Supplementary Figures and Tables

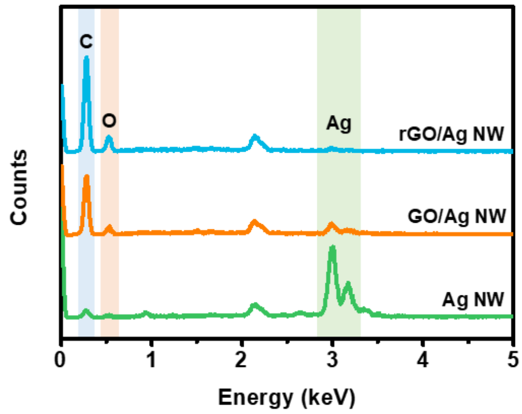

Fig. S1 The EDS spectra of the Ag NW, GO/Ag NW, and rGO/Ag NW films

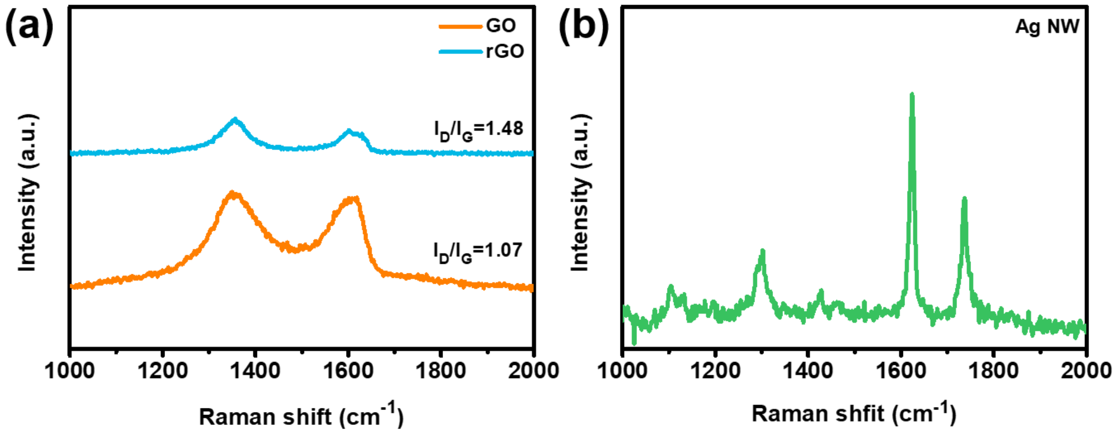

Fig. S2 Raman spectrum of **a** GO and rGO, **b** Ag NW

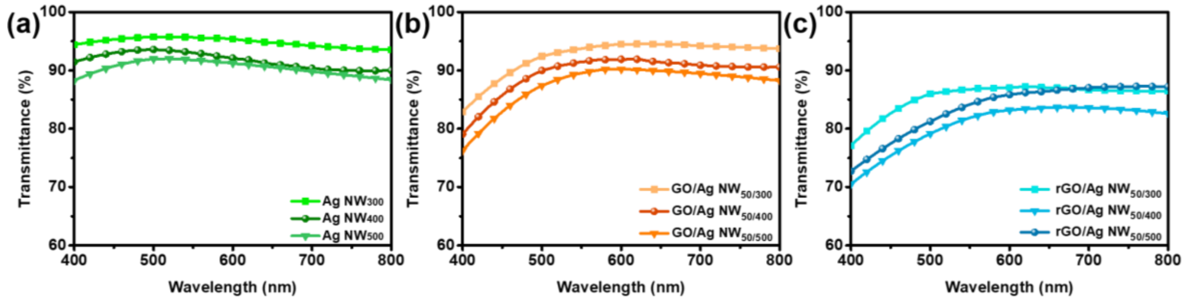

Fig. S3 Optical transmittance of **a** Ag NW, **b** GO/Ag NW, **c** rGO/Ag NW

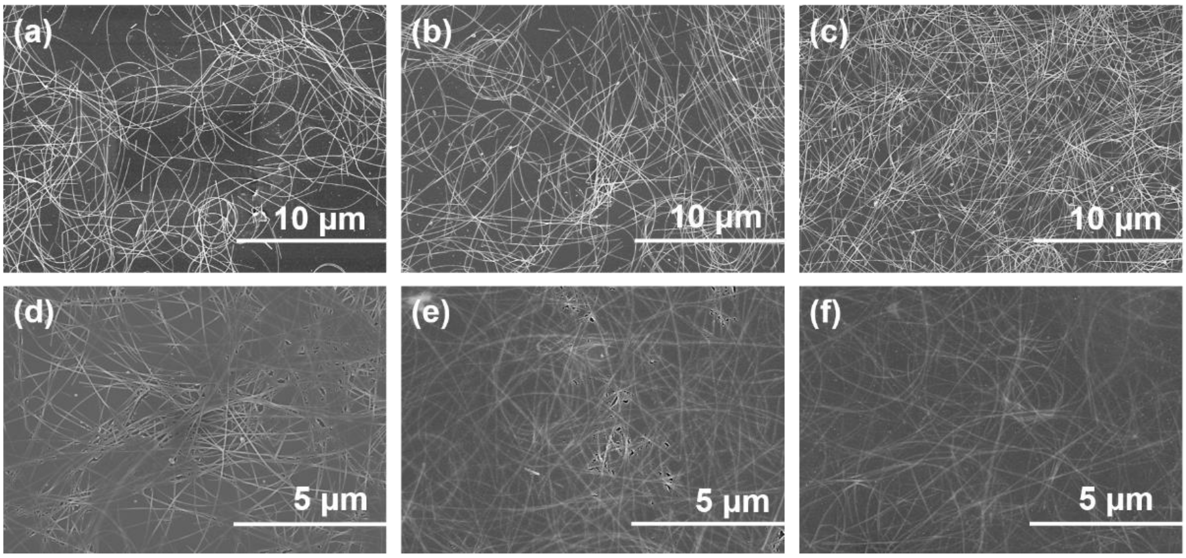

Fig. S4 SEM images **a** Ag NW_{300}, **b** Ag NW_{400}, **c** Ag NW_{500}. SEM images of the rGO/Ag NW film with various rGO area density of **d** 30 mg· m^{-2}, **e** 40 mg· m^{-2}, **f** 50 mg· m^{-2}

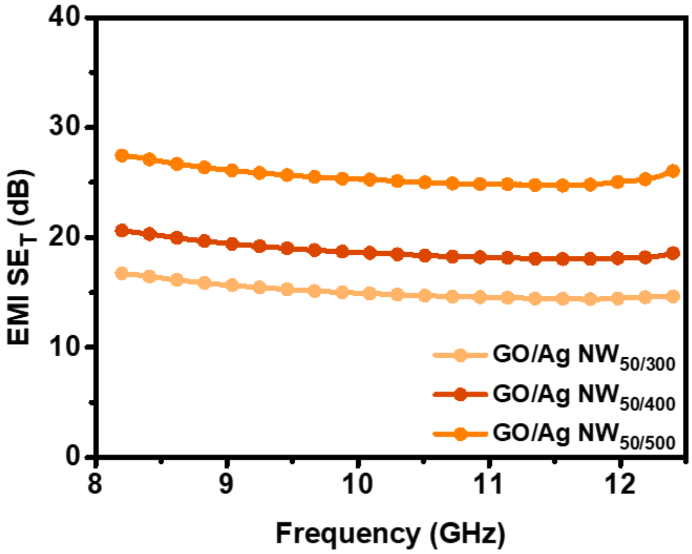

Fig. S5 EMI SE_T value of the GO/Ag NW films

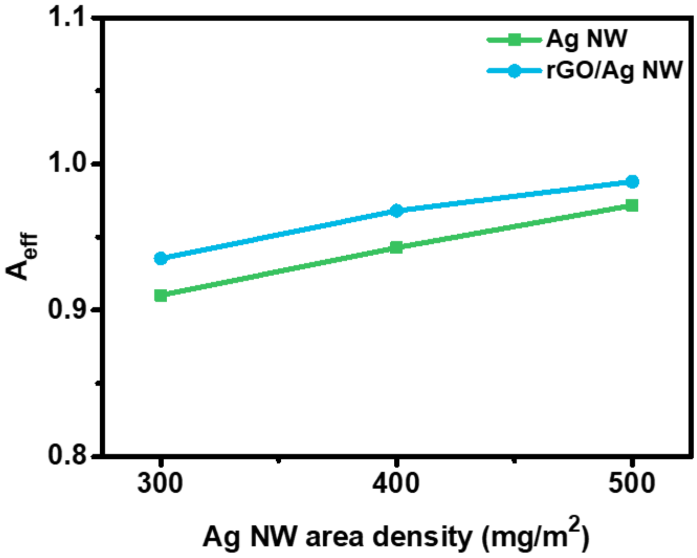

Fig. S6 A_{eff} of the Ag NW and rGO/Ag NW films

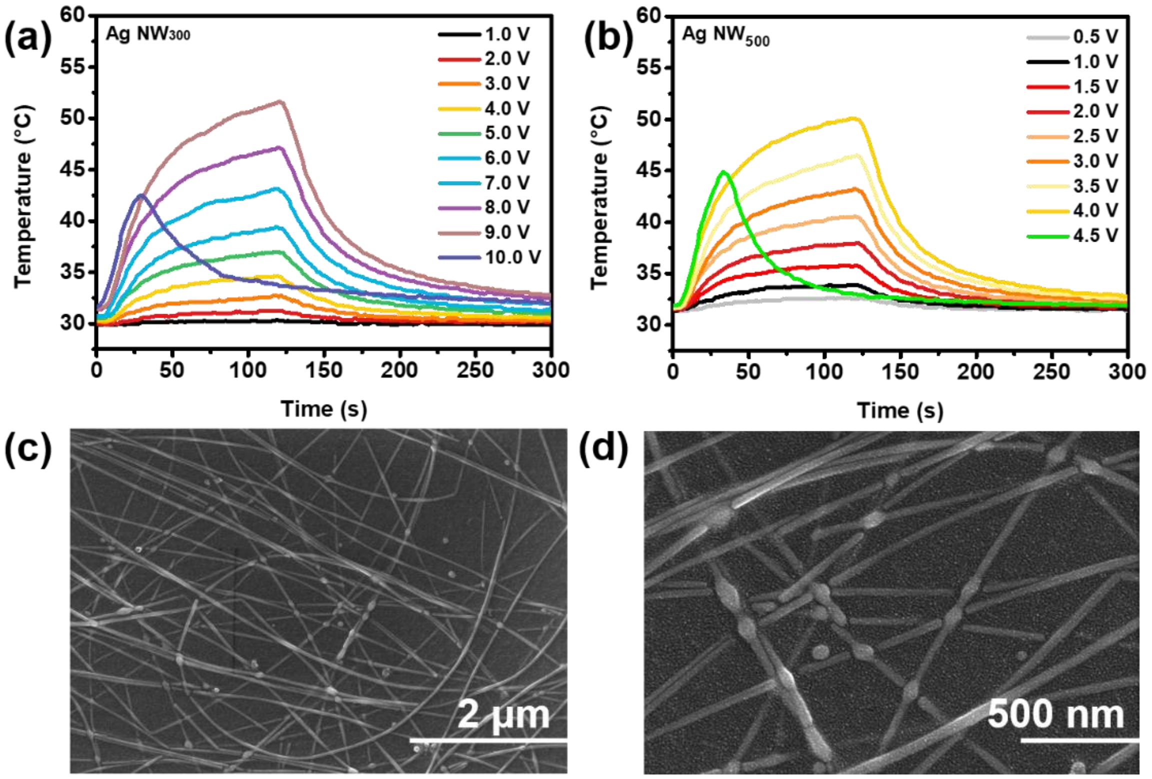

Fig. S7 Temperature-time profiles of the **a** rGO/Ag NW_{300}, **b** Ag NW_{500} at different applied voltages. **c, d** SEM images of the fractured Ag NW network
